# Geothermal Energy Impact Estimator: A software application for estimating the life-cycle environmental impacts of geothermal energy

**DOI:** 10.12688/openreseurope.15304.3

**Published:** 2023-06-27

**Authors:** Andrea Paulillo, Xiaofei Cui, Paul Brown, Alberto Striolo, Paola Lettieri

**Affiliations:** 1 Department of Chemical Engineering, University College London, London, WC1 E7JE, UK; 2 TWI Ltd., Cambridge, CB21 6AL, UK; 3 School of Chemical, Biological and Materials Engineering, University of Oklahoma, Norman, Oklahoma, 73019, USA

**Keywords:** Renewable energy, geothermal wells, Enhanced Geothermal Systems, carbon footprint, simplified models, allocation strategy, policy-making, decision-making.

## Abstract

Geothermal energy is a renewable source of base-load power that is expected to play an important role in the transition to a low-carbon economy. In this article, we introduce a novel software application – named Geothermal Energy Impact Estimator – which computes the environmental impacts, including carbon emissions, of existing or future geothermal plants, using the Life Cycle Assessment (LCA) methodology. The software application is user-friendly and was designed to be used by geothermal companies and policy makers. We provide two specific use cases of the software application that represent existing plants in Iceland and in the UK.

## Introduction

Geothermal is a renewable source of energy: it embodies the natural heat content of the Earth that dates back to its formation and that it is continuously renewed via radioactive decay. Unlike other renewable energy sources such as solar and wind, geothermal energy is independent of seasonal and climatic conditions, and therefore it can generate base-load power. This and other features, including a substantial theoretical potential for electricity generation ^1, 2^, make geothermal a promising energy source to expedite the decarbonisation of the power generation sector, and the transition to the low-carbon economy required to mitigate and when possible prevent long-term consequences of global warming ^3^. However, the contribution of geothermal energy to global electricity production remains limited: in 2020, the industry generated only 0.3% of global electricity generation from all sources and 1.2% from renewable sources ^4^. The International Energy Agency (IEA) projects the sector’s output to grow at an annual rate of 5% to 2024; but, according to the Agency, this is only about half of what is required to meet worldwide carbon neutrality by 2050 ^5^.

Nowadays, most of the geothermal installed capacity is represented by traditional geothermal power plants that use well-known technologies to convert thermal energy into electricity like dry steam, and single-/double-flash plants. The Geysers Complex in California (US) is the largest conventional geothermal field in the world, with a total electric capacity of ~1.5GW ^6, 7^. A more recent geothermal technology - known as Enhanced Geothermal Systems (EGS) – has attracted considerable interests. Whilst traditional geothermal plants take advantage of high-enthalpy hydrothermal reservoirs that are typically confined near geological plate boundaries, EGS harness geothermal energy in locations that lack reservoirs but that have higher-than-average thermal gradients; this is enabled via the development of “engineered” reservoir using stimulation techniques ^8^. The ability of EGS to extract heat in areas that lack water or sufficient permeability significantly extends the applicability of geothermal energy to vast areas of the planet. In Europe, efforts to develop EGS have focused in the Upper Rhine Valley (a region that extends across France, Germany and Switzerland): the first worldwide commercial-scale power plant was commissioned at Soultz-sous-Forêts in France ^9, 10^. In the United Kingdom, the United Downs Deep Geothermal Power (UDDGP) project is aiming to develop the country’s first commercial geothermal power plant, which relies on heat produced by the Cornish granites and exploits the natural permeability of a significant structural fracture zone ^11, 12^.

Life Cycle Assessment (LCA) is a standardised and widely adopted methodology to quantify the environmental impacts associated with a product throughout its life-cycle ^13, 14^. The life-cycle perspective and the consideration of a number of environmental issues enable identification of trade-offs and hot spots, thus providing a robust framework for decision support. The LCA methodology has been widely applied to compare the environmental performance, including carbon emissions, of disparate energy technologies (e.g. see 15, 16). Despite the standardised framework, the results of LCA studies on geothermal power generation show high variability; for example, the carbon footprint of geothermal-derived electricity stretches over two order of magnitudes, from ~5 and up to ~800 gCO _2_-eq./kWh ^17^. This variability is in part due to methodological choices like the definition of the system boundary, and in part to the fact that the environmental impacts strongly depend on site-specific conditions such as the composition of the geothermal fluid or the depth of the geothermal reservoir ^18, 19^, which in turn determine, for example, the magnitude and type of gaseous emissions or the structural requirements of geothermal wells. Notably, the latter aspect emphasizes the importance of collecting high-quality field data to reliably estimate the environmental footprint - arguably the most time-consuming phase of LCA.

In this article, we present a software application - named Geothermal Energy Impact Estimator (GEIE) – that predicts the life-cycle environmental impacts of geothermal plants for generation of electricity generation or for co-generation of electricity and heat. An earlier, non-peer reviewed version of this article is available as a Horizon 2020 deliverable report ^20^. The presented application has a twofold goal. First, it attempts to tackle the variability of LCA results due to methodological choices; notably, the variability of site-specific conditions and the importance of individual parameters has been investigated by Paulillo *et al*. ^21^, whose work represents the basis for the development of the software application presented in this article. Second, the application aims to provide a user-friendly tool designed for people not familiar with the LCA methodology; for instance, the application could be used by policy makers to support the development of energy policies, and by geothermal companies designing and operating geothermal power plants.

## Methods

### Implementation

Geothermal Energy Impact Estimator (GEIE) is a Microsoft Windows based application written using C# (see *Software availability* ^22^). The objective of the software application is the quantification of the retrospective or prospective life-cycle environmental impacts of geothermal energy: retrospectively, to assess the environmental performance of an existing plant, and prospectively, to predict that of a future plant.

The computational core of GEIE relies on two parametric models, named “full” and “simplified”; both are described in detail in 20. The “full” model is a comprehensive parametric model featuring 32 input parameters that quantifies the environmental impacts of electricity and, when applicable, thermal energy generation. The input parameters are colour-coded according to whether default values are provided and how uncertain they are. The “full” model is a modified version of the model presented in Paulillo *et al*. ^21^. The modified “full” model extends the application to cover the generation of thermal energy, including the case of co-generation (which is dealt with via allocation factors, as explained in the “Operation” sub-Section. The “simplified” model relies on a subset of influential input parameters (four for conventional and three for EGS plants), defined as those parameters that contribute the most to the variance of the “full” model. These parameters were identified by means of Global Sensitivity Analysis in 21; the resulting “simplified” model was presented in 23.

The “full” model is more accurate than the “simplified” model (provided that the input parameter values are accurate) but it requires more data to be collected. Both parametric models use detailed and validated life-cycle inventories obtained from the literature (see 20, 21) and from Ecoinvent ^24^, a commercial LCA database. GEIE computes impacts using in the environmental categories included in the Environmental Footprint 2.0 ^25^, which are reported in Table 1 ^1^.

**Table 1.** Environmental impact categories of the Environmental Footprint 2.0 (EF2.0) method, as implemented in Geothermal Energy Impact Estimator (GEIE).

Impact categories	Units	Acronym
Acidification, terrestrial and freshwater	Mole of H+ eq.	A
Climate change	kg CO2 eq.	CC
Eutrophication, freshwater	kg P eq.	Ef
Eutrophication, marine	kg N eq.	Em
Eutrophication, terrestrial	Mole of N eq.	Et
Ecotoxicity, freshwater	CTUe	ETf
Human toxicity, cancer effects	CTUh	HT-c
Human toxicity, non-cancer effects	CTUh	HT-nc
Ionising radiations	Bq U235 air-eq.	IR
Land use	Points	LU
Ozone depletion	kg CFC-11 eq.	OD
Respiratory inorganics	kg PM2.5 eq.	RI
Photochemical ozone formation	kg NMVOC eq.	POF
Resource use, fossils	MJ	RUe
Resource use, mineral and metals	kg Sb eq.	RUm
Resource use, water	m³ eq.	RUw

To develop a user-friendly application, we i) included input parameters expected to be available to geothermal companies, and ii) optimised the Graphical user interface (GUI) design to facilitate usage by those not familiar with LCA. We note that some parameters may not be available by for prospective studies; for examples, the amount of diesel consumed for drilling of wells would only be known after drilling is completed. For these parameters, users should input appropriate estimates based on e.g. data from similar sites or predicting models (e.g. 26 for diesel consumption). We envisage that the software can support policy makers in the development of energy policies and geothermal companies in designing and operating geothermal power plants.

### Operation

**Figure 1.**
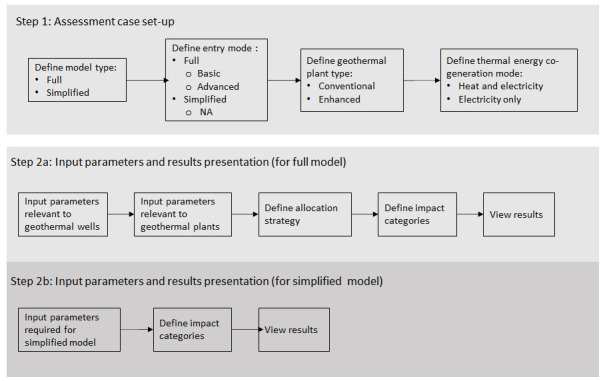
Overview of the GEIE (Geothermal Energy Impact Estimator) software application workflow.

**Figure 2.**
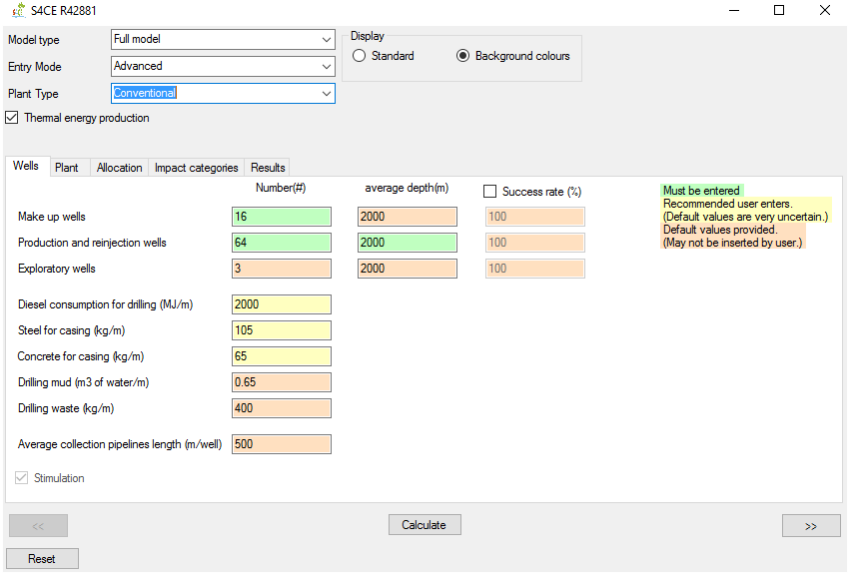
Case set-up and Wells tab of the GEIE (Geothermal Energy Impact Estimator).

The only requirement of the GEIE software application is .Net Framework 4.7.1 or later versions. Figure 1 shows the overall workflow of the software application, whilst Figure 2– Figure 4 and Figure 5 provide an outlook of the GUI of GEIE.

**Figure 3.**
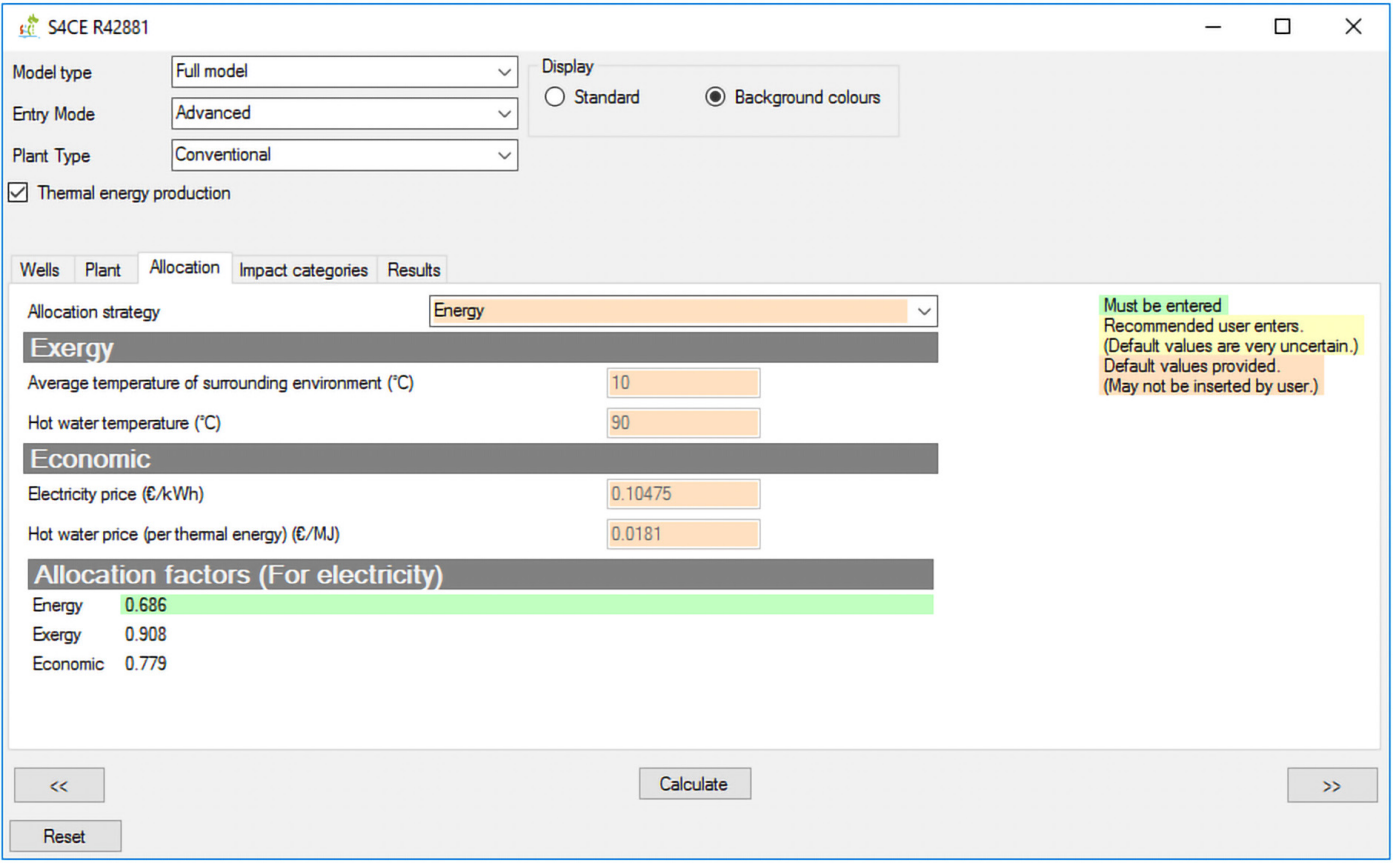
Allocation tab of the GEIE (Geothermal Energy Impact Estimator).

**Figure 4.**
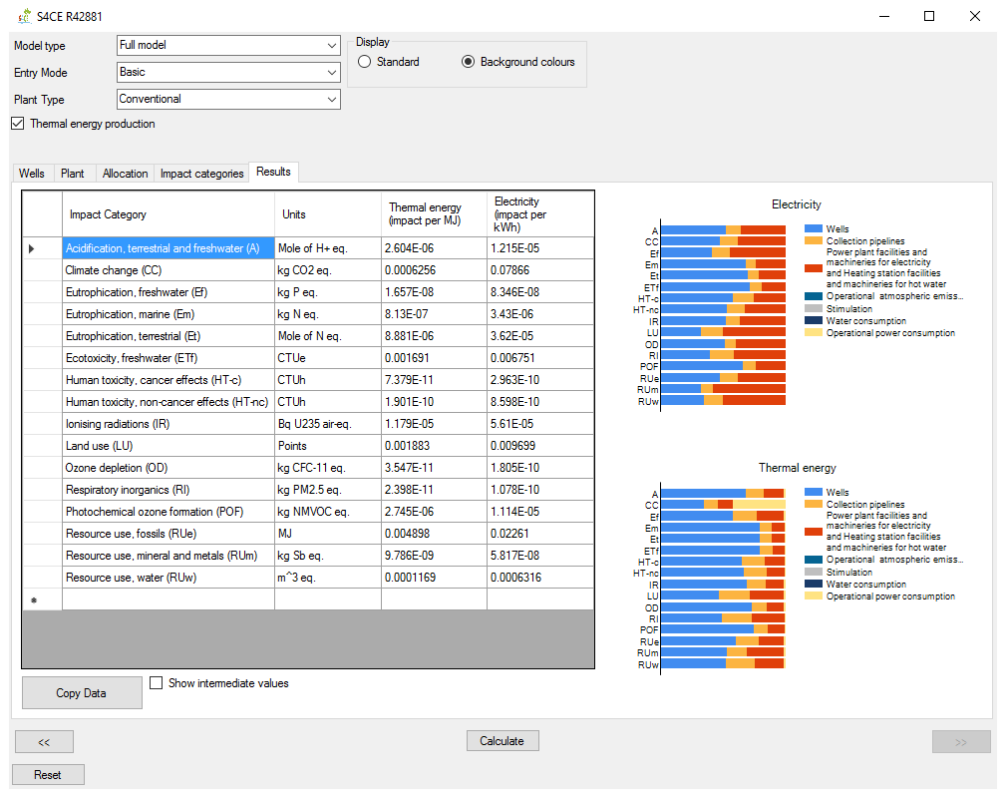
Results tab of the GEIE (Geothermal Energy Impact Estimator).

**Figure 5.**
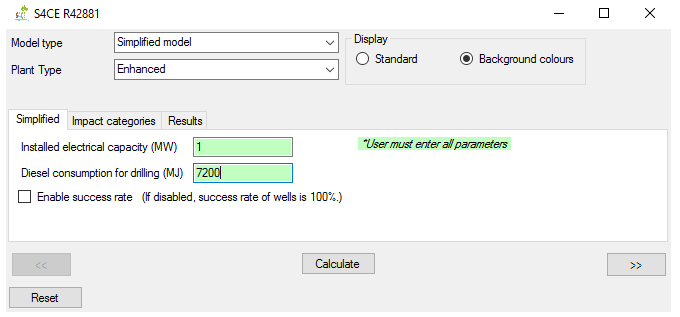
“Simplified” main tab of the GEIE (Geothermal Energy Impact Estimator).

The first step is the assessment case set-up. At the top of the interface shown in Figure 2, the User can choose between the “full” and the “simplified” model type. If the “full” model is selected, the User also needs to select the Entry Mode, i.e., “basic” or “advanced”, depending on the User’s preference on the level of control over all parameters. The “basic” Entry Mode only enables inserting values for the input parameters that are colour-coded in Green and in Yellow. The former identifies mandatory input parameters, whilst the latter those parameters that we recommend being provided by the User because the default values that we included are highly uncertain. The “advanced” Entry Mode allows to override input parameters for which default values are provided; these parameters are labelled in orange. Below the Entry Mode, the software application presents an additional drop-down menu that allows selection of the type of geothermal plant, i.e., conventional ^2^ and enhanced. Finally, the “thermal energy production” toggle box enables/disables the co-generation of heat and power; when the toggle box is deselected, the software assumes generation of electricity only.

The second step entails inserting values of the input parameters and viewing and/or exporting the results. If the “full” model is selected, the software features five tabs. The “Wells” ( Figure 2) and “Plant” tabs enable entering values for the input parameters that are relevant to the geothermal wells and plant respectively. The “Wells” tab also includes a toggle box that applies to enhanced geothermal plants only and that enables/disables hydraulic stimulation of geothermal wells. The “Allocation” tab ( Figure 3) is only relevant for the case of co-generation of heat and power: it allows selecting the strategy for allocating the environmental impacts between electricity and thermal energy, and, if the entry mode “advanced” is selected, also changing the values of the relevant parameters. When allocation is enabled, the activities that are partitioned include construction of wells and pipelines, and stimulation (if applicable). The remaining activities are allocated to the individual functions of electricity or thermal energy generation; more details are provided in 20. From the “Impact categories” tab the User can select the environmental categories for which the software will calculate the impact scores. Finally, the environmental impacts are reported in the “Results” tab ( Figure 4) as numerical values and graphically, as contribution analysis. The environmental impacts are reported per unit of electricity (in kWh), and per unit of heat (in MJ) when the relevant option is enabled. The “Results” tab also provides the option to export the numerical values of the environmental impacts, for example into an Excel spreadsheet.

The application’s operation for the model type “simplified” features three tabs. The main tab ( Figure 5) allows the User to insert the relevant parameters, which differs between conventional and enhanced geothermal plants. The main tab also includes a toggle box related to the parameter “success rate”, which is defined as the percentage of successfully drilled wells. When the toggle box is disabled, the model assumes a 100% success rate for all wells’ types; when it is enabled, the user can modify the success rate (the provided default value for primary wells is 72% ^23^). The option to enable/disable the “success rate” parameter is important because this parameters can in some cases considerably affect the LCA results, although it is not as significant as the other parameters of the “simplified” model ^21, 23^. The remaining two tabs for the ”simplified” model – “Impact categories” and “Results” – are similar to those for the “full” model.

## Use cases

We developed two specific use cases of the GEIE software application representing two geothermal plants: the Hellisheidi heat-and-power cogeneration plant located near Reykjavik, Iceland and the United Downs Deep Geothermal Power Project (UDDGP) in Cornwall, United Kingdom. For each use case, we report both input parameters and GEIE’s outputs (see also *Underlying data* ^27^). We note that the validity of the “full” and the “simplified” models have been analysed in detail in 21, 23; it is therefore outside the scope of the present article to discuss the results, including the differences between the outputs of the two models.

### Hellisheidi geothermal plant

Hellisheidi, the most recent geothermal project in Iceland, is the largest geothermal plant in the country and the sixth largest in the world by electric capacity ^7^. The environmental performance of Hellisheidi’s operation has been assessed in detail by Karlsdottir *et al*. ^28, 29^ and by Paulillo *et al*. ^17, 30^. In Table 2, we report the input parameters for the “full” model (“advanced” entry mode) for the Hellisheidi geothermal plant. The values are based on site-specific data collated by Karlsdottir *et al*. and reported in 28 and 17. In Table 3, we report the outputs of the GEIE software application. These are obtained using the “full” model with “advanced” entry mode, selecting “conventional” as plant type, enabling the co-production of thermal energy, adopting energy as allocation strategy, and selecting all environmental categories. Note that the table reports impacts for electricity and heat because the Hellisheidi plants co-generates both products and the relevant option in GEIE was enabled.

**Table 2.** Input parameters developed for the Hellisheidi geothermal power plant. These parameters are obtained from an open-access publication ^17^.

Parameter	Unit	Value
**Wells**		
**Number**		
Make up wells	#	16
Production and reinjection wells	#	64
Exploratory wells	#	0
**Average depth**		
Make up wells	m	2220
Production and reinjection wells	m	2220
Exploratory wells	m	0
Diesel (for drilling)	MJ/m	2262
Steel (for casing)	kg/m	100
Cement (for casing)	kg/m	40
Drilling mud	m3 of water	1.00
Drilling waste	kg/m	450
**Success rate**		
Production and Reinjection wells	-	1
Make-up wells	-	1
Exploratory wells	-	1
**Plant**		
Installed power, electricity	MW	303.3
Lifetime	years	30
Capacity factor	-	0.87
Auxiliary power	-	0.04
Installed power, thermal	MW	133
Heating station power consumption	kWh/s	0.40
Average collection pipelines length	m/well	500
Organic working fluid	kg/MWel	0
Cooling towers	#/MWel	0.023
Direct CO _2_ emissions	kg CO _2_/kWh	2.09E-02
Direct CH _4_ emissions	kg CH _4_/kWh	3.50E-05
**Stimulation**		
Number of wells to be stimulated	#	-
Water	m3 of water	-
Electricity	kWh/m3 of water	-
**Allocation**		
Average temperature of surrounding environment	°C	10
Hot water temperature	°C	90
Electricity price	kr/kWh	5.87
Hot water price (per thermal energy)	kr/MJ	0.708

**Table 3.** Outputs of GEIE (Geothermal Energy Impact Estimator) as applied to data for the Hellisheidi geothermal plant.

Category	Unit	Wells	Collection pipelines	Power plant/ Heating station facilities and machineries	Operational atmospheric emissions	Stimulation	Water consumption	Operational power consumption	Total
**Electricity (1 kWh)**									
Acidification, terrestrial and freshwater (A)	Mole of H+ eq./kWh	7.73E-06	1.43E-06	4.55E-06	0	0	0	0	1.37E-05
Climate change (CC)	kg CO2 eq./kWh	9.00E-04	2.55E-04	6.51E-04	3.33E-13	0	0	0	2.40E-02
Eutrophication, freshwater (Ef)	kg P eq./kWh	4.02E-08	1.18E-08	3.85E-08	0	0	0	0	9.05E-08
Eutrophication, marine (Em)	kg N eq./kWh	2.88E-06	2.85E-07	8.57E-07	0	0	0	0	4.02E-06
Eutrophication, terrestrial (Et)	Mole of N eq./kWh	3.12E-05	3.23E-06	8.14E-06	0	0	0	0	4.25E-05
Ecotoxicity, freshwater (ETf)	CTUe/kWh	5.87E-03	6.55E-04	1.36E-03	0	0	0	0	7.88E-03
Human toxicity, cancer effects (HT-c)	CTUh/kWh	1.97E-10	5.15E-11	7.75E-11	0	0	0	0	3.26E-10
Human toxicity, non-cancer effects (HT-nc)	CTUh/kWh	5.33E-10	1.24E-10	2.92E-10	0	0	0	0	9.48E-10
Ionising radiations (IR)	Bq U235 air-eq./ kWh	3.50E-05	6.49E-06	2.12E-05	0	0	0	0	6.27E-05
Land use (LU)	Pt/kWh	3.45E-03	1.72E-03	5.04E-03	0	0	0	0	1.02E-02
Ozone depletion (OD)	kg CFC-11 eq./kWh	1.15E-10	1.60E-11	7.34E-11	0	0	0	0	2.04E-10
Respiratory inorganics (RI)	kg PM2.5 eq./kW	4.76E-11	2.09E-11	4.64E-11	0	0	0	0	1.15E-10
Photochemical ozone formation (POF)	kg NMVOC eq./kWh	9.00E-06	1.16E-06	2.79E-06	5.31E-18	0	0	0	1.33E-05
Resource use, fossils (RUe)	MJ/kWh	1.30E-02	3.31E-03	9.01E-03	0	0	0	0	2.53E-02
Resource use, mineral and metals (RUm)	kg Sb eq./kWh	1.71E-08	5.69E-09	3.50E-08	0	0	0	0	5.77E-08
Resource use, water (RUw)	m³ eq./kWh	2.59E-04	9.79E-05	3.24E-04	0	0	0	0	6.81E-04
**Heat (1 MJ)**									
Acidification, terrestrial and freshwater (A)	Mole of H+ eq./kWh	2.15E-06	3.96E-07	4.27E-07	0	0	0	4.74E-08	3.02E-06
Climate change (CC)	kg CO2 eq./kWh	2.50E-04	7.09E-05	7.85E-05	0	0	0	8.29E-05	4.82E-04
Eutrophication, freshwater (Ef)	kg P eq./kWh	1.12E-08	3.29E-09	3.67E-09	0	0	0	3.13E-10	1.84E-08
Eutrophication, marine (Em)	kg N eq./kWh	8.00E-07	7.91E-08	8.36E-08	0	0	0	1.39E-08	9.76E-07
Eutrophication, terrestrial (Et)	Mole of N eq./kWh	8.66E-06	8.98E-07	9.30E-07	0	0	0	1.47E-07	1.06E-05
Ecotoxicity, freshwater (ETf)	CTUe/kWh	1.63E-03	1.82E-04	1.64E-04	0	0	0	2.73E-05	2.00E-03
Human toxicity, cancer effects (HT-c)	CTUh/kWh	5.47E-11	1.43E-11	1.17E-11	0	0	0	1.13E-12	8.19E-11
Human toxicity, non-cancer effects (HT-nc)	CTUh/kWh	1.48E-10	3.44E-11	2.80E-11	0	0	0	3.28E-12	2.14E-10
Ionising radiations (IR)	Bq U235 air-eq./ kWh	9.73E-06	1.80E-06	1.83E-06	0	0	0	2.17E-07	1.36E-05
Land use (LU)	Pt/kWh	9.59E-04	4.77E-04	5.34E-04	0	0	0	3.53E-05	2.01E-03
Ozone depletion (OD)	kg CFC-11 eq./kWh	3.18E-11	4.46E-12	4.96E-12	0	0	0	7.05E-13	4.20E-11
Respiratory inorganics (RI)	kg PM2.5 eq./kW	1.32E-11	5.81E-12	6.39E-12	0	0	0	3.97E-13	2.58E-11
Photochemical ozone formation (POF)	kg NMVOC eq./kWh	2.50E-06	3.23E-07	3.77E-07	0	0	0	4.60E-08	3.24E-06
Resource use, fossils (RUe)	MJ/kWh	3.60E-03	9.18E-04	1.01E-03	0	0	0	8.74E-05	5.61E-03
Resource use, mineral and metals (RUm)	kg Sb eq./kWh	4.74E-09	1.58E-09	2.94E-09	0	0	0	2.00E-10	9.46E-09
Resource use, water (RUw)	m³ eq./kWh	7.20E-05	2.72E-05	2.82E-05	0	0	0	2.35E-06	1.30E-04

### United Downs Deep Geothermal Power (UDDGP) project

The United Downs Deep Geothermal Power (UDDGP) represents the first deep geothermal power project in the UK. The project aims at harnessing the natural permeability of a significant structural fracture zone known as the Porthtowan Fault Zone ^11^. The geothermal wells were completed in 2019, and the power plant is expected to be operational in 2023. The environmental impacts of the UDDGP project were investigated by Paulillo *et al*. using a combination of primary data gathered on site and secondary data obtained from the literature ^12^; the full inventory data is publicly available ^31^. From these data, we report the input parameters for the “simplified” model in Table 4 and GEIE’s outputs in Table 5. Note that the outputs i) only refer to electricity (the “simplified” model does not include the case of co-generation) and ii) include both cases when the success rate is enabled (termed in the table “with success rate”) and disabled (“without success rate”).

**Table 4.** Input parameters developed for UGGDP (United Downs Deep Geothermal Power). These parameters are obtained from an open-access publication ^31^.

Parameter	Unit	Value
Installed electrical capacity	MW	1
Diesel consumption for drilling	MJ	7200

**Table 5.** GEIE (Geothermal Energy Impact Estimator) outputs as applied to data from UDDGP (United Downs Deep Geothermal Power).

Category	Unit	With Success rate	Without Success rate
Acidification, terrestrial and freshwater (A)	Mole of H+ eq./kWh	1.67E-03	1.29E-03
Climate change (CC)	kg CO _2_ eq./kWh	1.42E-01	1.09E-01
Eutrophication, freshwater (Ef)	kg P eq./kWh	2.70E-06	1.98E-06
Eutrophication, marine (Em)	kg N eq./kWh	6.27E-04	4.91E-04
Eutrophication, terrestrial (Et)	Mole of N eq./kWh	6.86E-03	5.37E-03
Ecotoxicity, freshwater (ETf)	CTUe/kWh	3.71E-01	2.66E-01
Human toxicity, cancer effects (HT-c)	CTUh/kWh	1.27E-08	9.13E-09
Human toxicity, non-cancer effects (HT-nc)	CTUh/kWh	3.56E-08	2.58E-08
Ionising radiations (IR)	Bq U235 air-eq./kWh	7.15E-03	5.53E-03
Land use (LU)	Pt/kWh	2.78E-01	2.08E-01
Ozone depletion (OD)	kg CFC-11 eq./kWh	2.66E-08	2.08E-08
Respiratory inorganics (RI)	kg PM2.5 eq./kW	4.62E-09	3.48E-09
Photochemical ozone formation (POF)	kg NMVOC eq./kWh	2.07E-03	1.60E-03
Resource use, fossils (RUe)	MJ/kWh	1.91E+00	1.46E+00
Resource use, mineral and metals (RUm)	kg Sb eq./kWh	1.33E-06	1.03E-06
Resource use, water (RUw)	m ^3^ eq./kWh	1.77E-02	1.32E-02

## Conclusions

In this article we presented a Microsoft Windows based software application - named Geothermal Energy Impact Estimator (GEIE) - which can be used to quantify the environmental impacts of existing or future geothermal plants, including both conventional and enhanced technologies. The computational core of the application relies on two parametric models, termed “full” and “simplified”. The models, which have been developed and validated elsewhere, rely on life-cycle inventories obtained from the literature and from commercial LCA databases. Because the software application is user-friendly, it should be easily implemented by policy makers, consultants, and energy companies. The workflow of GEIE is straightforward, consisting in a two-step process; the first being the definition of the assessment case set-up, and the second the insertion of the input parameter values and the viewing and/or exporting of the results. We have provided two specific use cases of GEIE as applied to two existing geothermal plants: Hellisheidi, a conventional co-generation plant in Iceland, and the United Downs Deep Geothermal Power (UDDGP) project in the UK. The details concerning the environmental impacts of these plants are available in the open literature, and have been referenced throughout the article.

## Ethics and consent

No ethical approval or consent were required.

## Data Availability

Zenodo: GEIE use cases. https://doi.org/10.5281/zenodo.7333572 ^27^. This project contains the following underlying data: HSD_IN.xlsx (input parameters developed for the Hellisheidi geothermal power plant. These parameters are obtained from an open-access publication ^17^). HSD_OUT.xlsx (outputs of GEIE as applied to data for the Hellisheidi geothermal plant). UGGDP_IN.xlsx (input parameters developed for UGGDP. These parameters are obtained from an open-access publication ^31^). UGGDP_OUT.xlsx (GEIE outputs as applied to data from UDDGP). Data are available under the terms of the Creative Commons Attribution 4.0 International license (CC-BY 4.0).

## Software availability

Source code available from: https://github.com/paul-brown-twi/GeothermalEnergyImpactEstimator/tree/v1.1

Note: the .exe file to install the software can be downloaded from the GitHub repository, under Releases.

Archived source code at time of publication: https://doi.org/10.5281/zenodo.7267704 ^22^

Licence: MIT
