# Supplementary material for: Geothermal Energy Impact Estimator: A software application for estimating the life-cycle environmental impacts of geothermal energy
Source: Open Res Eur. 2023 Jun 27;3:2. Originally published 2023 Jan 6. [Version 3] doi: 10.12688/openreseurope.15304.3 (PMC10445808; doi:10.12688/openreseurope.15304.3)
Supplement: Peer review history

# Peer review: Reviewer response for version 2

**DOI:** 10.21956/openreseurope.17259.r31841

**Published:** 2023-06-14

**Authors:** Neil Burnside

**Affiliations:** 1 University of Strathclyde, Glasgow, Scotland, UK

Paper does a good job of describing the software and providing guidance on its use. It looks like the authors have addressed previous reviewer comments. I believe it is is fit for purpose when considering the journal scope but recommend the authors address the, mostly minor, comments below. 

 The sentence, “ *The ability to extract heat in the absence of water or sufficient permeability significantly extends the applicability of geothermal energy to vast areas of the planet”, *is out of place here without further qualification. It’s wedged between sentences regarding EGS, which primarily aims to create sufficient permeability and circulate water. Suggest make more relevant (e.g., by discussing single well / closed loop systems) or removing completely.

*“In the United Kingdom, the United Downs Deep Geothermal Power (UDDGP) project is developing the first commercial geothermal power plant, which relies on heat produced by the Cornish granites and exploits the natural permeability of a significant structural fracture zone”. *Some information is presented as factual here when I don’t believe this is the case. May depending on use of ‘commercial’, but the Eden Project is at a similar stage so in practice may prove to be the first commercial plant in the UK (also note ‘UK’ should be added here- sentence as written suggests that UDDGP would be the first commercial plant in the world). Until circulation between wells has been achieved the claim that this system ‘exploits the natural permeability of a significant structural fracture zone’ is unproven. Suggest re-writing sentence as follows: “In the United Kingdom, the United Downs Deep Geothermal Power (UDDGP) project is **aiming to develop** the **UK’s** first commercial geothermal power plant, which relies on heat produced by the Cornish granites and **will aim to exploit** the natural permeability of a significant structural fracture zone”. *Note* I see similar sentence is worded more appropriately in a later section.

*“The geothermal wells were completed in 2019, and the power plant is expected to be operational in 2022*”. This article was first published in Jan 2023 and last updated in May 2023. This sentence should also be updated as the expected year of operation is prior to publication of the paper. Suggest authors check if  plant is currently operational or not and provide an updated year for expected operation if it’s the latter.

*“When the toggle box is disabled, the model assumes a 100% success rate for all wells’ types; when it is enabled, the model assumes an average success rate lower than 100% (e.g. 72% for primary wells)”. *Does the ‘e.g.’ imply this ratio can be altered by the user? Or are there a set of defined valued baked into the tool? User alteration capability here would seem to have more use. Other studies suggest success rates of closer to 50%.

*“The details concerning the environmental impacts of these plants are available in the open literature, and have been referenced”. *The details may be in other referenced publications but would be useful for readers of this guide to see a summary, even if a paragraph or two, of what the results mean for the examples provided. This would help emphasize the utility of the tool and benefit intended users who may be time-pressured and not have the capacity to review several different articles. Addition of a summary of what is 'good' and 'bad' impact results (e.g. in comparison with other energy generation options) would help place geothermal in context for policy makers, consultants,

 and energy companies (the intended users of the tool).

Is the rationale for developing the new software tool clearly explained?

Yes

Is the description of the software tool technically sound?

Yes

Are sufficient details of the code, methods and analysis (if applicable) provided to allow replication of the software development and its use by others?

Yes

Is sufficient information provided to allow interpretation of the expected output datasets and any results generated using the tool?

Partly

Reviewer Expertise:

Geothermal, hydrogeology, low carbon energy

I confirm that I have read this submission and believe that I have an appropriate level of expertise to confirm that it is of an acceptable scientific standard.

# Response

**Published:** 2023-06-20

**Authors:** Andrea Paulillo

**Affiliations:** University College London, UK

Many thanks for reviewing our article. Below are short responses to the comments provided:

- To our understanding, EGS does extend the applicability of geothermal energy because it enables extracting  heat in areas that lack water or sufficient permeability.  We have clarified the above sentence as follows: 
  “The ability of EGS to extract heat in areas that lack water or sufficient permeability significantly extends the applicability of geothermal energy to vast areas of the planet.”
- We have modified the sentence accordingly.
- We have corrected the year the plant is expected to be operational to 2023.
- We believe that describing the LCA results falls outside the scope of this paper, which aims at describing a tool rather than the environmental performance of geothermal energy.

# Peer review: Reviewer response for version 2

**DOI:** 10.21956/openreseurope.17259.r31249

**Published:** 2023-06-14

**Authors:** Astu Sam Pratiwi

**Affiliations:** 1 Renewable Energy Systems, Institute for Environmental Sciences (ISE), Section of Earth and Environmental Sciences, University of Geneva, Geneva, Switzerland

The modification made in Version 2 of this article has addressed the major concerns associated to Version 1. 

 Point 5 in the initial feedback has not been addressed, however this is understandable as it would require a significant additional work. It is however recommended to have uncertainty range (or confident interval) included in future GEIE update. 

 The reviewer deemed, to the best of her knowledge, that this article is good to index. Reiterating previous impression, this work contributes to the advancement of scientific research in the geothermal industry by promoting LCA thinking and providing quantitative assessment.

Is the rationale for developing the new software tool clearly explained?

Yes

Is the description of the software tool technically sound?

Yes

Are sufficient details of the code, methods and analysis (if applicable) provided to allow replication of the software development and its use by others?

Partly

Is sufficient information provided to allow interpretation of the expected output datasets and any results generated using the tool?

Partly

Reviewer Expertise:

Life cycle assessment modelling of geothermal power plant and heat plant in non volcanic regions, maintenance and operation of EGS power plant. I did not review the codes as this is not my area of expertise.

I confirm that I have read this submission and believe that I have an appropriate level of expertise to confirm that it is of an acceptable scientific standard.

# Peer review: Reviewer response for version 1

**DOI:** 10.21956/openreseurope.16546.r30773

**Published:** 2023-04-06

**Authors:** Astu Sam Pratiwi

**Affiliations:** 1 Renewable Energy Systems, Institute for Environmental Sciences (ISE), Section of Earth and Environmental Sciences, University of Geneva, Geneva, Switzerland

This article's scientific significance is underscored by its addressing of the need for prospective Life Cycle Assessment (LCA) in conventional and enhanced geothermal systems. By promoting LCA thinking in the development of energy systems, it contributes to the advancement of scientific understanding in this field. Furthermore, this research offers an accessible approach for comprehensively evaluating the environmental impacts of geothermal systems, providing a forward-looking perspective. However, to further enhance the article's contribution, it is suggested that the authors address the following points.

1. Elaborate what activities are being allocated in the co-generation scenario. I suppose the heat exchangers for heating production is accounted as the burden of only thermal energy.
2. Diesel consumption in GJ per m has been reported as an influential parameter. However, I am not convinced that  it is the most appropriate input parameter for prospective LCA as the knowledge of diesel consumption per meter is normally known after drilling is accomplished. It is derived from other parameters such as hole size, geological formation/condition, and depth. You are invited to refer to Table A1 in the Supplementary data of https://doi.org/10.1016/j.geothermics.2020.101988 to see the possibility of predicting the diesel consumption from other input parameters.
3. Similarly, the capacity of geothermal plant depends on the temperature, flowrate (which itself depends on injectivity and productivity index) and the phase of geothermal fluid. However, the relationships can be very complex and defining the capacity as input parameter is acceptable.
4. The environmental impact of operational power consumption should also vary depending of installation country unless it is assumed that it will use part of the electricity generated from geothermal energy
5. Would be great if the level of accuracy of simple and full modes can be represented with visualization of uncertainty range. E.g, the values of impacts in simple mode will show higher uncertainty range than if full mode is selected.
6. I could not find a step by step installation for the user who wants to use the calculator here. Has it been provided in different location? It needs clarification.
  Further, the following bold words need correction:
7.  Whilst traditional geothermal plants take advantage of high-enthalpy hydrothermal reservoirs that are typically confined near geological plate boundaries, EGS harness geothermal energy in locations that lack reservoirs but that have higher-than-average thermal gradients; **this is enable(d)** via the development of “engineered” reservoir using stimulation techniques.
8. Life Cycle Assessment (LCA) is a standardised and widely adopted methodology to quantify the environmental impacts associated with a product throughout its life-cycle. The life-cycle perspective and the consideration of a number of environmental issues **enables (no s)** identification of trade-offs and hot spots, thus providing a robust framework for decision support.

Is the rationale for developing the new software tool clearly explained?

Yes

Is the description of the software tool technically sound?

Yes

Are sufficient details of the code, methods and analysis (if applicable) provided to allow replication of the software development and its use by others?

Partly

Is sufficient information provided to allow interpretation of the expected output datasets and any results generated using the tool?

Partly

Reviewer Expertise:

Life cycle assessment modelling of geothermal power plant and heat plant in non volcanic regions, maintenance and operation of EGS power plant. I did not review the codes as this is not my area of expertise.

I confirm that I have read this submission and believe that I have an appropriate level of expertise to confirm that it is of an acceptable scientific standard, however I have significant reservations, as outlined above.

# Response

**Published:** 2023-04-19

**Authors:** Andrea Paulillo

**Affiliations:** University College London, UK

Many thanks for reviewing our article. We are glad this was appreciated. Please find below point-by-point responses to the comments raised:

1. Thanks for raising this point, which is critical. We have added the following text to clarify how allocation has been implemented:
  “When allocation is enabled, the activities that are partitioned include construction of wells and pipelines, and stimulation (if applicable). The remaining activities are allocated to the individual functions of electricity or thermal energy generation; more details are provided in 20.”
2. This is an important point. One could argue that other parameters like the hole size and/or conditions of the reservoir may not (or only partially) be known in advance for prospective LCAs. We chose diesel consumption as an input parameters because the underlying models in this study (refs 20, 21 and 23) are based on a detailed review of LCA studies available in the scientific literature, where diesel consumption is nearly always reported. 
  We agree that some parameters may not be known in advance for prospective studies, but this should not limit the use of our software because users may estimate these parameters from e.g. the literature of predicting models like those that you mention. To clarify this point, we have added the following sentence:
  “We note that some parameters may not be available by for prospective studies; for examples, the amount of diesel consumed for drilling of wells would only be known after drilling is completed. For these parameters, users should input appropriate estimates based on e.g. data from similar sites or predicting models (e.g.  32 for diesel consumption).”
3. We agree
4. Yes: our model assumes that the operational power consumption will be met using part of the electricity generated by the plant.
5. This is a very interesting point. It is not applicable in the current software but we will consider it as a future improvement. 
6. We have added a note to clarify how to use the software:
  “the .exe file to install the software can be downloaded from the GitHub repository, under Releases.”

# Peer review: Reviewer response for version 1

**DOI:** 10.21956/openreseurope.16546.r30689

**Published:** 2023-02-16

**Authors:** Giampaolo Manfrida

**Affiliations:** 1 Department of Industrial Engineering, University of Florence, Florence, Italy

The article proposed by Paulillo et al. is about a tool (“Geothermal Energy Impact Estimator”) for estimating the environmental impacts through Life Cycle Assessment of geothermal plants. The article is well written, and correctly covers all the necessary aspects.

 The overall assessment of the article is that its indexing should be approved.

 Some comments are given below to improve both communication and the tool output potential:

- The advice to make the concept of variability clearer for non-experts is to point out that site-specific conditions and the technology used for the plant can determine the presence or absence of gas emissions to air and considerable differences in the number of wells. Both are elements that have a considerable environmental impact
- The text states that “ *the full model is a modified version of the model presented in Paulillo et al.21 …”. *For a better understanding, it would be  important to specify briefly what modifications have been made.
- The text often mentions 'conventional plants', I would recommend to make it clear whether 'conventional' means exclusively flash plants or flash and ORC and Hybrid  plants.
- Due to the use of the Environmental footprint 2.0 methodology, I would recommend to add a short comment on the uncertainty of the results for the categories of Toxicity (ecotoxicity and human toxicity), which can determine uncertainty in the final  interpretation of the results.
- In the case of Co-generation, considering that there is high variability in the results obtained with different allocation factors, I believe that the authors could also indicate the most correct solution from a product point of view, i.e. to choose an exergy-based  allocation principle.
- the quality of Figure 3 (screenshot capture) should be improved, particularly the grey characters on a light grey background (good for a screen, not for publication).
- In the case of cogeneration, it is stated in the text that environmental impacts refer to 1 kWh electricity and 1 MJ of heat. Was the possibility of reporting the results in terms of exergy considered? I think it could be an added value to the consistency of the results obtained.
- In the text, there is a parameter called 'success rate', which defines in generic terms the success rate of the well drilling. The concept should be extended: the reader should be aware of what is the meaning.
- For a scientific approach - as well as for decision-making, it is important to be able to compare different plants (obviously considering that the geological/resource context is very different). Moreover it would be good for decision-making to be able to compare the environmental footprint of a geothermal energy project with possible alternatives for Renewable Energy at regional scale (e.g. solar - PV or thermal conversion - and wind energy at least). It is quite common that such alternatives exist and this would be very useful for local policy-makers as well as for Energy Communities.

**Is the rationale for developing the new software tool clearly explained?**

 The article clearly explains in the introduction the reason for the development of the software and in fact wants to solve the variability of LCA results for geothermal plants, which are very dependent on the case study and the methodology used, and also wants to make the application of these analyses easy even for non-experts in the field.

**Is the description of the software tool technically sound?**

 The description of the tool is appropriate to the article, and uses technical terms related to both LCA analysis and the geothermal plants in an understandable manner.

**Are sufficient details of the code, methods and analysis (if applicable) provided to allow replication of the software development and its use by others?**

 The methodology used is clear and easy to understand in all its aspects. The reader clearly follows the steps in the different cases of analysis (simplified, full). There is not enough detail of the code or analysis in this article. But in the text you will find all the specific references indicating where to find the information needed to correctly interpret the analysis as it has already been made public. In addition, the reference to access the code via github is also given.

**Is sufficient information provided to allow interpretation of the expected output datasets and any results generated using the tool?**

 In order to support non-expert readers, the results obtained from two case studies, a combined heat and power plant and an EGS plant, are given. In addition, references are given for the two case studies of previously published papers that cover the analysis in detail. This gives a complete insight into the interpretation of the outputs obtained.

Is the rationale for developing the new software tool clearly explained?

Yes

Is the description of the software tool technically sound?

Yes

Are sufficient details of the code, methods and analysis (if applicable) provided to allow replication of the software development and its use by others?

Partly

Is sufficient information provided to allow interpretation of the expected output datasets and any results generated using the tool?

Yes

Reviewer Expertise:

Renewable Energy, Geothermal Power Plants, LCA, Exergy, Exergo-Economic and Exergo-Environmental Analyses, Advanced Energy Systems, Sustainability of Energy COnverion and Utilization

I confirm that I have read this submission and believe that I have an appropriate level of expertise to confirm that it is of an acceptable scientific standard.
